# Association between antepartum depressive symptoms and prenatal care utilization and milestones: a retrospective cohort study

**DOI:** 10.1186/s12884-025-07489-0

**Published:** 2025-04-03

**Authors:** Minyoung Jang, Malini Ramaiyer, Sarah Olson, Kristin Voegtline, Cybill Esguerra

**Affiliations:** 1 Department of Obstetrics and Gynecology, Johns Hopkins University School of Medicine, 600 North Wolfe Street, Baltimore, MD USA; 2 Biostatistics, Epidemiology, and Data Management Core, Johns Hopkins University, Baltimore, MD USA; 3 Division of General Pediatrics, Department of Pediatrics, Johns Hopkins University School of Medicine, Baltimore, MD USA

**Keywords:** Depression, Mental health, Prenatal care, Glucose tolerance testing, Group B streptococcus testing

## Abstract

**Background:**

To evaluate the relationship between antepartum depressive symptoms and prenatal care utilization and completion of prenatal care milestones, namely, routine screening tests and vaccinations in pregnancy.

**Methods:**

This retrospective cohort includes pregnant individuals who completed first or second trimester screening using the Edinburgh Postnatal Depression Scale (EPDS) between 2020 and 2021. Baseline characteristics were compared between individuals who screened positive versus negative for depression. Multivariable logistic regression models were used to estimate odds of completing third trimester prenatal care milestones, including sexually transmitted infection screening, glucose tolerance testing, group B streptococcus testing, and tetanus toxoid, reduced diphtheria toxoid, and acellular pertussis (Tdap) vaccination.

**Results:**

Of 718 individuals who completed the EPDS in early pregnancy, 85 (11.8%) screened positive for depressive symptoms. A greater proportion of these individuals were younger (median age 28 vs. 31 years, *P =*.034), unpartnered (*P* =.005), publicly insured (*P* =.013), had a history of substance use (*P* <.001) or prior psychiatric diagnosis (*P* <.001), and entered prenatal care at later gestational ages (median 12.3 vs. 10.7 weeks, *P* =.002) than those who screened negative. Although there were no significant differences in total prenatal care visits attended between groups (*P* =.123), the adjusted odds of completing third trimester milestones including glucose tolerance testing (OR [95% CI] 0.60 [0.36–0.98]) and group B streptococcus testing (OR 0.56 [0.33–0.95]) were lower among individuals who screened positive for depressive symptoms. Less than half of individuals at risk for depression who were referred to mental health services successfully completed consultations during pregnancy.

**Conclusions:**

Pregnant individuals who screen positive for depressive symptoms in early pregnancy comprised a socially vulnerable group at risk of missing third trimester prenatal care milestones. Mental health screening and integrated interventions across the perinatal continuum may help mitigate the current treatment gap for patients in need of mental health services during pregnancy.

## Background

Perinatal depression is a common obstetric complication that can lead to serious consequences for parent and child. Perinatal depression can occur during pregnancy or within one year postpartum, with an estimated global pooled prevalence of 11.9% [1]. Existing literature on perinatal depression predominantly focuses on the impact of postpartum depression on longer-term maternal, neonatal, and childhood outcomes. A 2019 systematic review [2] of 122 studies suggests that postpartum depression is detrimental to the personal wellbeing of mothers, leading to strained social relationships [3], increased substance use [4], and persistent depression [5]. Research also links postpartum depression to stressful mother-child interactions (e.g., difficulty breastfeeding) [6] and impaired cognitive and psychosocial development of children [7].

Postpartum depression is strongly associated with mood symptoms prior to and during pregnancy [8], yet research is limited regarding depression in the antepartum period. Some studies have assessed the relationship between prenatal depressive symptoms and health care utilization during pregnancy, demonstrating an increased risk of late establishment of prenatal care [9], fewer overall prenatal visits [10], more non-scheduled visits [11], and higher medical costs [12]. Fewer studies have examined the relationship between maternal depression and the completion of key prenatal care milestones, namely routine screening tests and vaccinations, that may be important predictors of perinatal outcomes [13]. Understanding potential patterns of suboptimal prenatal care utilization and missed milestones may reveal specific opportunities for targeted intervention among individuals with antepartum depression.

In this study, we aimed to assess the relationship between depressive symptoms and the utilization of routine prenatal and non-routine health care services during pregnancy, as well as the completion of certain third trimester prenatal care milestones. We also described patterns of referral and engagement with mental health services among our cohort of individuals at risk for depression.

## Methods

### Study design

This was a retrospective cohort study performed in the Johns Hopkins Medicine (JHM) health system.

### Setting and participants

Inclusion criteria were pregnant individuals of all ages who established prenatal care at three JHM clinical sites, completed depression screening during the first or second trimester of pregnancy using the Edinburgh Postnatal Depression Scale (EPDS), and delivered a live birth between January 1, 2020 and December 31, 2021. Individuals with an unknown number of prenatal visits were excluded from this study. Two of the clinical sites (Nelson Clinic and the Johns Hopkins Outpatient Center) are obstetric clinics located within Johns Hopkins Hospital in East Baltimore. The third site (Green Spring Station) is a standalone ambulatory center located approximately 12 miles north of Johns Hopkins Hospital in Baltimore County.

### Variables

The EPDS is a self-reported, 10-item questionnaire that has been translated into 50 different languages and excludes constitutional symptoms of depression that can be commonplace in pregnancy [14]. Although the EPDS was originally developed to be administered postnatally, its use has been validated antenatally [15, 16]. During the study period, JHM clinics transitioned to a protocol implementing routine antenatal depression screening using the EPDS, in accordance with American College of Obstetricians and Gynecologists (ACOG) guidelines at that time to screen patients for depression at least once during the perinatal period [17]. For individuals with multiple EPDS scores recorded during the first two trimesters of pregnancy, we considered their highest EPDS score for analysis. For individuals with multiple deliveries in the study time frame, we selected their earlier pregnancy for analysis for standardization purposes.

Individuals and cases were classified according to exposure to risk for depression. Individuals in the control group were defined as those who scored < 10 on the EPDS. Cases of individuals at risk for depression were defined as those who scored ≥ 10 on the EPDS and/or answered affirmatively to item #10 (suicidal ideation) on the questionnaire. Various threshold scores have been recommended for a positive depression screen; a cutoff of 10 was selected for this study based on clinician experience in the JHM system and ACOG literature [17].

The primary outcome of this study was prenatal care utilization, defined as number of prenatal care visits attended, treated as both a discrete and dichotomous variable in analysis. As a dichotomous variable, prenatal care utilization was defined as low (< 8 visits) versus high (≥ 8 visits). Traditionally, the recommended amount of prenatal care visits averages around 12–14 total visits [18]. That said, the World Health Organization recommends attending eight or more prenatal care visits to reduce perinatal mortality, indicating that less than eight visits may reflect insufficient utilization [19]. Of note, the study time frame overlaps with the first two years of the Coronavirus disease 2019 (COVID-19) pandemic– due to limitations of our bulk extraction data collection methods, in-person and telemedicine encounters could not be distinguished, therefore both were included in analysis.

Secondary outcomes of this study included: (1) Non-routine health care utilization, defined as number of Labor & Delivery (L&D) triage visits attended, analyzed as both a discrete and dichotomous variable with utilization defined as low (< 2 visits) versus high (≥ 2 visits). Any L&D triage encounters documented within 72 hours of a patient’s delivery date were excluded from analysis as these visits were likely in the setting of presenting for labor. (2) Completion of third trimester prenatal care milestones, namely screenings for sexually transmitted infections (STI), including human immunodeficiency virus (HIV) and syphilis, gestational diabetes mellitus (GDM), group B streptococcus (GBS), as well as tetanus toxoid, reduced diphtheria toxoid, and acellular pertussis (Tdap) vaccination, each dichotomized as yes or no. Third trimester milestones were specifically selected for analysis to support a temporal association between screening positive for depressive symptoms in early pregnancy and care completed later in pregnancy.

### Data sources

For our primary variables of interest, we performed bulk data extraction from Epic Electronic Medical Record system [20]. When determining chronic maternal, pregnancy-related, and fetal complications, these data were extracted from Epic using ICD-10 codes. For historical pregnancy-related conditions and chronic maternal conditions, ICD-10 codes prior to the start of the index pregnancy were assessed. For pregnancy-related conditions, ICD-10 codes after the start of the index pregnancy and prior to delivery were assessed.

We also performed a manual chart review of a subset of cases who screened positive for antepartum depression to collect data on referrals placed and completed for social work, therapy, and psychiatric services. At clinical sites included in this study, social work referrals are routinely placed for those with positive depression screens, typically at an initial prenatal intake performed by nursing, unless declined by the patient, or at later time points in pregnancy as clinically indicated. All individuals specifically followed through the Center for Fetal Therapy, which cares for patients with pregnancies affected by complex fetal conditions, are seen by social work. Social workers and/or other obstetric providers subsequently refer patients to therapy and/or psychiatry within or outside of the JHM system as clinically indicated, unless they are already engaged with these services or they decline referral. Consultations were considered complete based on visit documentation.

### Study size

In our retrospective analysis, we anticipated a minimum cohort size of 200 participants that had completed the EPDS during the antenatal period. This sample size would yield 80% power at a 0.05 significance level to detect lower odds of prenatal care utilization among those who screen positive for elevated depressive symptoms (estimated at 25%) relative to those who do not screen positive (estimated at 75%) (effect size range of odds ratios (ORs) = 4.53 to 12.08).

### Statistical methods

To compare baseline demographic and pregnancy-related characteristics between study groups as well as assess crude associations between our exposure and primary and secondary outcomes, categorical variables were analyzed using Pearson’s chi-squared or Fisher’s Exact test, and discrete variables were analyzed using Student’s t or Wilcoxon Rank-Sum test. Logistic regression models were used to estimate ORs of completing prenatal milestones. Adjusted models were further constructed, controlling for maternal and obstetric covariates that were judiciously selected due to the relatively small sample size of our study: a key socioeconomic indicator (insurance status), a key health engagement indicator (gestational age at first prenatal visit), and a key pregnancy history indicator (gravidity). Statistical significance was defined as *P* <.05. Descriptive statistics were also reported to describe patterns of mental health service referral for individuals who screened positive for depression. Analyses were performed using SAS 9.4 [21].

## Results

**Fig. 1:**
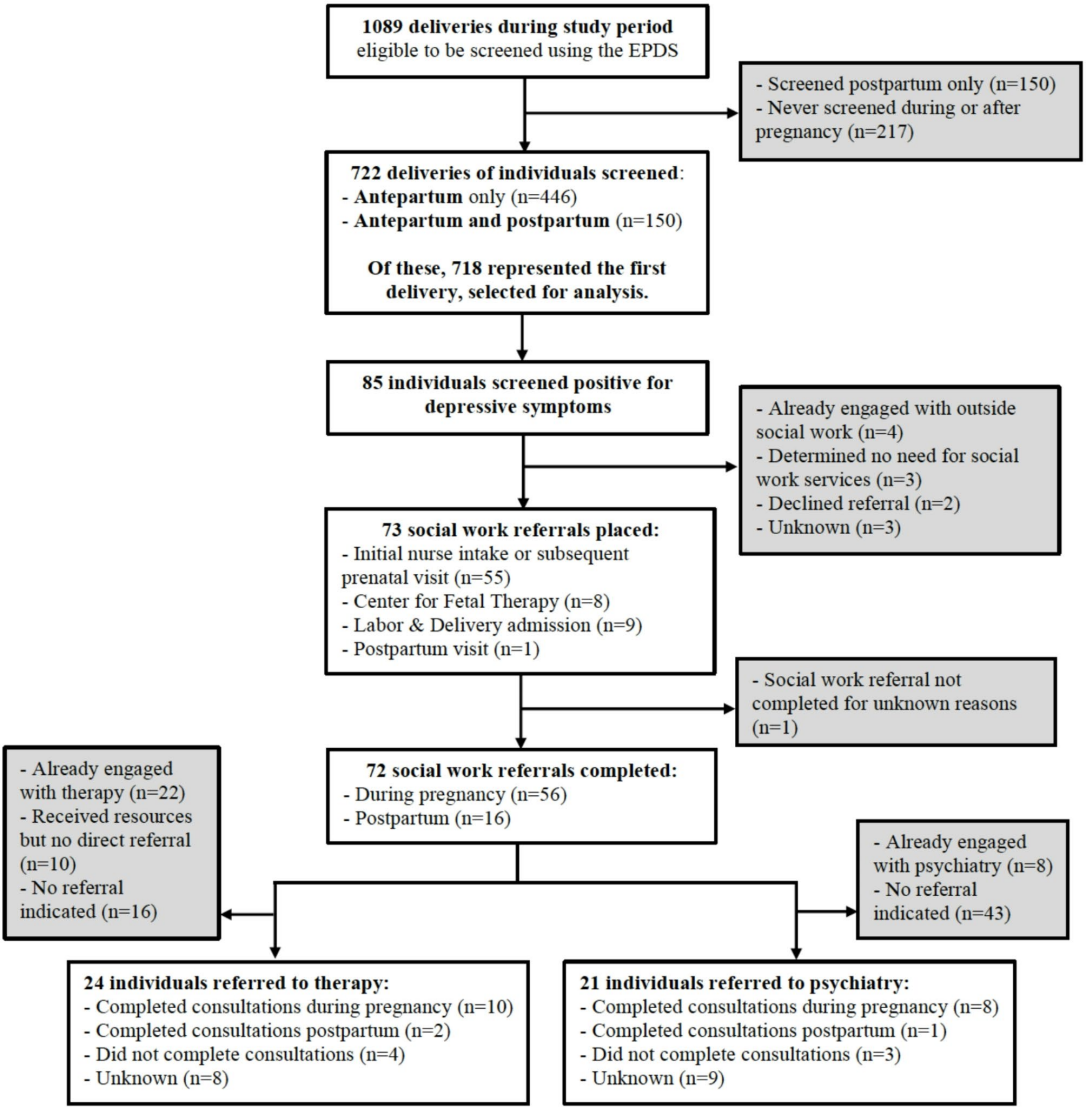
Depression screening using the Edinburgh Postnatal Depression Scale (EPDS) by perinatal stage, study inclusion, and mental health resource referral pathways

Of 1089 deliveries that occurred during the study period, 446 individuals (41.0%) were screened for depressive symptoms using the EPDS antepartum only, 276 (25.3%) were screened antepartum and postpartum, 150 were screened postpartum only (13.8%) at obstetric postpartum visits, and 217 (19.9%) were never screened during or after pregnancy. Among the 722 deliveries of individuals who were screened antepartum, 718 represented the first delivery for each individual during the study period and were selected for analysis (Fig. 1).

Of 718 individuals who completed the EPDS in the first or second trimester of pregnancy, 85 (11.8%) screened positive for depressive symptoms. A greater proportion of these individuals were younger at first prenatal visit (median age 28 vs. 31 years, *P* =.034), unpartnered (not married or in domestic partnership) (*P* =.005), publicly insured (*P* =.013), and had a history of substance use (documented prior use of tobacco or other non-alcoholic drugs in the electronic health record) (*P* <.001) or documented prior psychiatric diagnosis (*P* <.001) (Table 1). There were no statistically significant differences in race, ethnicity, highest education level, or employment status between the two cohorts.

**Table 1:** Baseline characteristics of individuals who screened positive versus negative for depressive symptoms on the Edinburgh Postnatal Depression Scale

	EPDS positive	EPDS negative	Total	
**Characteristic**	*n* = 85 (11.8)	*n* = 633 (88.2)	*n* = 718	*P*-value
Age at first prenatal visit, years	28 (24–34)	31 (26–34)	30 (26–34)	0.034*
Race				
Black or African American	45 (52.9)	275 (43.4)	320 (44.6)	0.183
White or Caucasian	31 (36.5)	254 (40.1)	285 (39.7)	
None of the above	9 (10.6)	104 (16.4)	113 (15.7)	
Ethnicity				
Not Hispanic or Latino	80 (94.1)	597 (94.5)	677 (94.4)	0.804
Hispanic or Latino	5 (5.9)	35 (5.5)	40 (5.6)	
Highest education level				
High school or less	51 (60.7)	437 (69.0)	488 (68.1)	0.124
Some college or more	33 (39.3)	196 (31.0)	229 (31.9)	
Marital status				
Partnered	38 (44.7)	385 (60.8)	423 (58.9)	0.005*
Unpartnered	47 (55.3)	248 (39.2)	295 (41.1)	
Employment				
Employed	57 (67.1)	434 (68.6)	491 (68.4)	0.780
Unemployed/Student	28 (32.9)	199 (31.4)	227 (31.6)	
Insurance status				
Private	37 (43.5)	376 (59.4)	413 (57.5)	0.013*
Government	47 (55.3)	254 (40.1)	301 (41.9)	
Other	1 (1.2)	3 (0.5)	4 (0.6)	
Substance Use				
Tobacco use	15 (17.7)	33 (5.2)	48 (6.7)	< 0.001*
Alcohol use	9 (10.6)	73 (11.5)	82 (11.4)	0.797
Drug use	15 (17.7)	27 (4.3)	42 (5.9)	< 0.001*
Psychiatric History				
Any history of psychiatric diagnosis	55 (64.7)	229 (36.2)	284 (39.6)	< 0.001*

EPDS, Edinburgh Postnatal Depression Scale

Data are *n* (%) or median (interquartile range)

*P <.05

Individuals at risk for depression were more likely to start prenatal care at a median gestational age of 12.3 weeks, approximately two weeks later than those who screened negative for depression on the EPDS (*P* =.002) (Table 2). Individuals in the EPDS positive group also had a higher median gravidity (*P* =.012) and were more likely to have a history of prior abortion (*P* =.009). While the frequency of pregnancy related complications was relatively even between cohorts, 80% of pregnancies among individuals at risk for depression were affected by chronic maternal complications compared to 72.8% of pregnancies among individuals who screened negative on the EPDS (*P* =.159). In addition, 49.4% of pregnancies among individuals who screened positive were affected by fetal complications (i.e. decreased fetal movement, fetal growth restriction, or other complex fetal conditions and anomalies, including eight patients receiving care through the Center for Fetal Therapy), compared to 26.4% of pregnancies among their counterparts who screened negative (*P* <.001).

**Table 2:** Maternal and pregnancy-related characteristics of individuals who screened positive versus negative for depressive symptoms on the Edinburgh Postnatal Depression Scale

Characteristic	EPDS positive	EPDS negative	Total	*P*-value
	*n* = 85 (11.8)	*n* = 633 (88.2)	*n* = 718	
Maternal characteristics				
Gestational age at first prenatal visit, weeks	12.3 (9.9–22.1)	10.7 (9.3–14.4)	10.9 (9.4–15.1)	0.002*
Advanced maternal age (> 35 years)	15 (17.7)	108 (17.1)	123 (17.1)	0.893
Pre-pregnancy BMI, kg/m^2^	27.7 (23.6–33.8)	27.1 (22.8–32.8)	27.3 (22.9–32.8)	0.383
Gravidity and parity				
No. gravida	3 (2–4)	2 (1–4)	2 (1–4)	0.012*
No. para	1 (0–2)	1 (0–2)	1 (0–2)	0.221
History of prior abortion	50 (58.8)	277 (43.8)	327 (45.5)	0.009*
Complications				
Any chronic maternal complications	68 (80.0)	461 (72.8)	529 (73.7)	0.159
Any pregnancy-related complications	48 (56.5)	370 (58.5)	418 (58.2)	0.728
Any fetal complications	42 (49.4)	167 (26.4)	209 (29.1)	< 0.001*

EPDS, Edinburgh Postnatal Depression Scale; BMI, body mass index

Data are *n* (%) or median (interquartile range)

*P <.05

There was no statistically significant difference in the total number of routine prenatal care visits attended by individuals who screened positive versus negative on the EPDS, which was a median of 11 visits for both groups (*P* =.314) (Table 3). Dichotomizing utilization of prenatal care as high versus low, 23.5% of individuals who screened positive on the EPDS had low prenatal care utilization compared to 16.8% of those who screened negative, however this difference was not found to be statistically significant (*P* =.123). We also did not demonstrate differences in the distribution of prenatal care visits attended by trimester (data not presented in table). With regard to non-routine care utilization, individuals at risk for depression visited L&D triage a median of twice during pregnancy, compared to once among those who screened negative for depression. This difference trended toward statistical significance (*P* =.088). Of note, higher EPDS scores were positively correlated with increased number of triage visits (*r* =.103, *P* =.006).

**Table 3:** Care utilization and completion of prenatal milestones among individuals who screened positive versus negative for depressive symptoms on the Edinburgh Postnatal Depression Scale

Variable	EPDS positive	EPDS negative	Total			*P*-value
	*n* = 85 (11.8)	*n* = 633 (88.2)	*n* = 718			
Total number of visits						
Prenatal care	11 (8–13)	11 (9–13)	11 (9–13)			0.314
L&D triage	2 (1–4)	1 (0–3)	1 (0–3)			0.088
Low vs. high care utilization						
Prenatal care visits						
< 8	20 (23.5)	106 (16.8)	126 (17.6)			0.123
≥ 8	65 (76.5)	527 (83.3)	592 (82.5)			
L&D triage visits						
< 2	38 (44.7)	341 (53.9)	379 (52.8)			0.112
≥ 2	47 (55.3)	292 (46.1)	339 (47.2)			
Correlation between visit counts and EPDS score						
Prenatal care visits	*r* =.049, *P* =.659	*r*=-.058, *P* =.144	*r*=-.053, *P* =.160			NA
L&D triage visits	*r* =.212, *P* =.051	*r* =.057, *P* =.151	*r* =.103, *P* =.006*			NA
Third trimester prenatal care milestones						
Tdap vaccination	39 (45.9)	272 (43.0)	311 (43.3)			0.611
STI screening	53 (62.4)	386 (61.0)	439 (61.1)			0.807
GDM screening	46 (54.1)	424 (67.0)	470 (65.5)			0.019*
GBS screening	60 (70.6)	532 (84.0)	592 (82.5)			0.002*

EPDS, Edinburgh Postnatal Depression Scale; L&D, Labor & Delivery; NA, not applicable; Tdap, tetanus toxoid, reduced diphtheria toxoid, and acellular pertussis; STI, sexually transmitted infection; GDM, gestational diabetes mellitus; GBS, group B streptococcus

Data are *n* (%) or median (interquartile range) unless otherwise indicated

*P <.05

There were relatively similar rates of completing Tdap vaccination and STI screening between EPDS positive and negative groups (Table 3). In contrast, the odds of completing GDM screening through glucose tolerance testing (OR [95% CI] 0.48 [0.30–0.77]) and GBS screening (OR 0.46 [0.27–0.76]) were statistically significantly lower among individuals who screened positive for depressive symptoms. These results remained significant after adjusting for insurance status, gestational age at first prenatal visit, and gravidity (GDM screening: aOR 0.60 [0.36–0.98]; GBS screening: aOR 0.56 [0.33–0.95]).

Among the subgroup of 85 patients who screened positive for depressive symptoms, social work referrals were placed for 73 (85.9%) individuals (Fig. 1). Of these, nearly all (98.6%) completed social work consultations. Of 72 individuals who met with social workers, 24 (33.3%) were referred to therapy, 22 (30.6%) were already engaged with therapists, 10 (13.9%) received mental health resources but were not directly referred to therapy, and 16 (22.2%) did not have an indication for referral. Of 24 individuals who were referred to therapy, 10 (41.7%) completed initial consultations with therapists during pregnancy, two (8.3%) completed consultations postpartum, four (16.7%) did not complete consultations, and the remainder (33.3%) had unknown outcomes (Fig. 1). In addition, social workers referred 21 (29.2%) individuals to psychiatry, eight individuals (11.1%) were already engaged with psychiatrists, and 43 (59.7%) did not have an indication for referral. Of 21 individuals who were referred to psychiatry, eight (38.1%) completed initial consultations with psychiatrists during pregnancy, one (4.8%) completed a consultation postpartum, three (14.3%) did not complete consultations, and the remainder (42.9%) had unknown outcomes. These results are presented in detail in Fig. 1.

## Discussion

In this retrospective cohort study, we did not demonstrate significant associations between screening positive for depressive symptoms in early pregnancy and total prenatal care visit attendance. We did, however, reveal novel findings that pregnant individals at risk of depression have lower odds of completing key third trimester prenatal milestones, specifically GDM and GBS screening, despite similar overall utilization of prenatal care. Higher EPDS scores were also positively correlated with an increased number of L&D triage visits, signifying a possible relationship between severity of depressive symptoms and use of non-routine health care services during pregnancy. While most individuals at risk for depression in early pregnancy were successfully engaged with social work, less than half of individuals ultimately completed referrals to therapy or psychiatry during pregnancy, highlighting a treatment gap between patients in need of mental health services and those that actually receive them.

Our lack of findings regarding prenatal care attendance contrast with existing studies that suggest an association between antepartum depression and inadequate prenatal care utilization, often defined according to the Kotelchuck Adequacy of Prenatal Care Utilization Index, which incorporates the month prenatal care begins and the total number of visits attended [9, 11, 22–24]. Although individuals who screened positive for depression in our study entered prenatal care approximately two weeks later than their counterparts who screened negative, the median gestational age of initiating care still fell within the first trimester and the median number of visits attended still reached approximately 80% of the recommended standard using either WHO (≥ 8 visits) or more traditional definitions (12–14 visits) [18, 19]. Our inconsistent results may be explained by higher rates of both chronic maternal and fetal complications among individuals at risk for depression, which is corroborated in other literature [25], which may necessitate and/or motivate increased prenatal care visit attendance among this cohort. The study period also overlaps with the early years of the COVID-19 pandemic. While the pandemic led to major disruptions in care for many women [26, 27], it is also possible that the transition to telemedicine actually increased access to care for certain populations through the use of technology [28, 29]. These findings remain relevant as obstetric practices across the country continue to adopt hybrid telehealth models and consider how to leverage these platforms to reach socially vulnerable groups including those at risk for depression in early pregnancy.

With regard to non-routine health care utilization, our finding that higher EPDS scores in the first or second trimester were positively correlated with an increased number of L&D triage visits during pregnancy aligns with other limited studies on this topic [11, 30]. Grajkowski et al. (2017) demonstrated that women with an EPDS score > 11 at their initial prenatal visit were more likely to engage in “super-utilization” of acute medical care, defined as four or more unscheduled medical visits during pregnancy and the first eight weeks after delivery [30]. While the median of two triage visits among individuals who screened positive for depression in this study fell below this “super-utilization” threshold, it is worth noting that our data did not account for emergency department visits or triage visits at L&D units outside of the JHM system so may represent an underestimate. Although greater prevalence of chronic maternal and/or fetal complications may, once again, confound the relationship between depressive symptoms and emergent health care utilization, identifying those at risk of over-using non-routine services early in pregnancy may help in better fulfilling their unmet medical and social needs while encouraging more efficient use of health care resources.

Notably, our study fills a gap in existing literature regarding antepartum depression and completion of prenatal care milestones, as we revealed that individuals who screen positive for depressive symptoms have lower odds of completing GDM and GBS screening in the third trimester. We initially posited that poor completion of milestones may be explained by low prenatal care attendance, however we did not find differences in total number of prenatal visits between our study groups, as discussed above. Patients may have perceived greater barriers to the screening processes for GDM and GBS compared to Tdap vaccination or STI testing, especially in the context of the COVID-19 pandemic. Some patients may have declined waiting one hour in the laboratory after consuming the glucose solution for GDM screening due to increased health care setting exposure. As our study demonstrated, individuals who screen positive for depressive symptoms in pregnancy are more likely to be younger, unpartnered, publicly insured, and have a history of substance use, underscoring the bidirectional relationship between mental health and social determinants of health that can further influence one’s ability to fulfill prenatal care milestones. To date, only one other study by Ben-Sheetrit et al. (2020) has reported on similar outcomes, revealing elevated risk for non-compliance with oral glucose tolerance test screening in pregnant individuals with depression as they under-utilized tests perceived to be for the wellbeing of the mother and over-utilized tests perceived to be for the wellbeing of the fetus (e.g., alpha-fetoprotein to detect neural tube defects) [13]. Understanding individual level reasons for not completing certain prenatal care screenings as well as the impact of incomplete third trimester testing in this setting on longer-term maternal or neonatal outcomes is outside the scope of this study.

Finally, we highlight opportunities for improvement in our depression screening and mental health resource referral pathways. In our study, close to 20% of pregnant individuals were never screened for depression perinatally, and only a quarter of patients were screened both antepartum and postpartum. In June 2023, ACOG released updated clinical practice guidelines that now recommend screening pregnant individuals for depression at the initial prenatal visit, later in pregnancy, and at postpartum visits [31]. Early and repeated screening across the perinatal continuum is crucial for identifying and treating depression expediently in pregnancy [32, 33]. However, successful connection to mental health treatment after individuals screened positive for depressive symptoms remained challenging in our cohort, despite commendably high rates of initial social work referral and consultation which are attributable to integrated social work services at most clinical sites included in this study. Despite a majority of completed social work consultations, less than half of individuals referred to therapy or psychiatry ultimately engaged with these services during pregnancy. One systematic review found that with screening alone, under a quarter of individuals with depression detected in outpatient perinatal care settings will be connected to treatment [34]. The need to schedule yet another visit for mental health services layers an additional burden on pregnant individuals who must navigate a complicated health care system and long wait times for appointments while carrying the physical and emotional stressors of both pregnancy and depression. One promising solution described in the literature is perinatal collaborative care, integrating medical, mental health–therapy and psychiatry–as well as social services into obstetric clinical settings to remove barriers to treatment of mental health disorders in pregnancy [35].

Limitations of this study include response bias, given the EPDS is a self-reported questionnaire and mental health may represent a sensitive topic for many individuals. Data are also limited by inability to extract certain information from Epic. Some patients completed print EPDS questionnaires, rather than electronic, either by request or because they did not have the MyChart electronic health record application downloaded at the time of this study– print questionnaires were scanned and uploaded to patients’ charts in a manner such that they could not be extracted for the purposes of this study. It is also difficult to represent potential confounding effects introduced by other factors related to the COVID-19 pandemic in our adjusted models. For example, although we have controlled for insurance status as a proxy for broader social determinants of health, acute changes in an individual’s economic circumstances during the pandemic may have impacted their ability to complete prenatal care. Individual circumstances that may necessitate stricter quarantine measures, such as sick family members or high risk occupations, may have also affected our studied outcomes. Lastly, data are missing on completed therapy or psychiatry referrals that took place outside of the JHM health system, which may lead to underestimation of completed consultations.

## Conclusions

In this study, pregnant individuals who screened positive for depressive symptoms in early pregnancy comprised a socially vulnerable group at risk of over-utilizing emergent health care services and missing third trimester prenatal care milestones despite adequate prenatal care visit attendance. More research is needed to understand the impact of missed milestones on perinatal outcomes. Depression screening at the initial prenatal visit and across the perinatal continuum may allow for earlier identification of these individuals at risk for depression and more timely intervention. Importantly, multidisciplinary approaches may help improve access to mental health care and close treatment gaps for this population.

## Data Availability

The datasets used and/or analyzed during the current study are available from the corresponding author on reasonable request.

## Abbreviations

ACOG: American College of Obstetricians and Gynecologists
COVID-19: Coronavirus disease 2019
EPDS: Edinburgh Postnatal Depression Scale
GBS: Group B beat-hemolytic streptococcus
GDM: Gestational diabetes mellitus
HIV: Human Immunodeficiency Virus
JHM: Johns Hopkins Medicine
L&D: Labor and Delivery
STI: Sexually transmitted infection
Tdap: Tetanus toxoid, reduced diphtheria toxoid, and acellular pertussis
